# Targeting ulcerative colitis by suppressing glucose uptake with ritonavir

**DOI:** 10.1242/dmm.036210

**Published:** 2018-11-21

**Authors:** Henrika Jodeleit, Omar Al-Amodi, Janina Caesar, Christina Villarroel Aguilera, Lesca Holdt, Roswitha Gropp, Florian Beigel, Matthias Siebeck

**Affiliations:** 1 Department of General-, Visceral-, Transplantation- and Vascular Surgery, University Hospital, LMU Munich, Nussbaumstr. 20, 80336 Munich, Germany; 2 Department of Laboratory Medicine, Institute of Laboratory Medicine, University Hospital, LMU Munich, 81377 Munich, Germany; 3 Department of Medicine II, University Hospital, LMU Munich, Marchioninistr. 15, 81377 München, Germany

**Keywords:** Ulcerative colitis, Metabolic switch, Glucose transporter, Ritonavir, NOD-scid *IL-2Rγ*^null^, Mouse model, Inflammation

## Abstract

Glucose is the preferred source of energy in activated inflammatory cells. Glucose uptake into the cell is ensured by a family of glucose uptake transporters (GLUTs), which have been identified as off-target molecules of the HIV protease inhibitor ritonavir. In this study, we examined the effect of ritonavir on inflammation *in vitro* and *in vivo*. Peripheral blood mononuclear cells (PBMCs) were activated with anti-CD3 in the presence or absence of ritonavir and analyzed by flow cytometric analysis. Frequencies of CD4+ cells were significantly affected by ritonavir (CD69+ *P*=3E-05; CD134 *P*=4E-06; CD25+ *P*=E-07; central memory *P*=0.02; effector *P*=6E-03; effector memory *P*=6E-05). To corroborate that inflammation has a metabolic effect *in vivo*, a mouse model was used that is based on immunocompromised NOD-scid *IL-2Rγ*^ null^ mice reconstituted with PBMCs from patients with ulcerative colitis (UC). Inflammation had a significant effect on amino acid (AA) levels (Glu *P*=1E-07, Asp *P*=1E-04). Principal component analysis (PCA) discriminated between unchallenged and challenged groups. Finally, the efficacy of ritonavir was tested in the same mouse model. Dependent variables were clinical and histological scores, frequencies of human leukocytes isolated from spleen and colon, and levels of AA in sera of mice. Mice benefited from treatment with ritonavir as indicated by significantly decreased colon (*P*=7E-04) and histological (*P*=1E-04) scores, frequencies of M2 monocytes (CD14+ CD163; *P*=0.02), and Glu levels (*P*=2E-05). PCA discriminated between control and challenged groups (*P*=0.026). Thus, inhibition of glucose uptake might be a promising therapeutic intervention point for active UC.

## Abstract (precis)

**Summary:** Insights into immuno-metabolism open up new avenues for therapeutic intervention. The study presented here suggests that inhibition of glucose suppresses inflammation in a mouse model of ulcerative colitis.

## INTRODUCTION

The energy supply of inflammatory cells relies on three sources: glycolysis, oxidation of lipids, and amino acid (AA) metabolism. In homeostasis, when the major task of inflammatory cells is the maintenance of tolerance, lipids are the preferred source as lipid oxidation is the most efficient albeit slowest pathway to generate ATP (for a review, see O'Neill et al., 2016). The response to an assault, however, requires the immediate activation, proliferation and differentiation of inflammatory cells, their migration to sites of inflammation, and expression of cytokines, growth factors and chemokines. These processes demand prompt energy supply, which is met by a metabolic switch from lipid oxidation to glycolysis to ensure swift ATP generation and the synthesis of biosynthetic intermediates, albeit at the expense of efficiency. Therefore, the dependence on glycolysis might offer an Achilles' heel of inflammatory cells.

This aspect is of great interest for treatment of chronic inflammatory diseases. Although great therapeutic improvement has been made with the approval of highly effective biologicals like anti-TNFα antibodies (infliximab, adalimumab and certolizumab), the anti-integrin-α4β7 antibody vedolizumab and the anti-IL12/23 antibody ustekinumab, many patients respond insufficiently to these drugs or develop a secondary loss of response and require additional or different treatments. Metabolic profiling of patients with ulcerative colitis (UC) and Crohn's disease (CD) in comparison to non-diseased individuals corroborated the assumption that, in these diseases, glycolysis is the preferred metabolic pathway (Dawiskiba et al., 2014; Bjerrum et al., 2017). Hence, targeting glycolysis might open up entirely novel treatment options for UC or CD. In addition to metabolites related to glycolysis, these studies have shown that, in sera of UC and CD patients, levels of AAs were either up [glutamic acid (Glu)] or down [histidine (His)] (Hisamatsu et al., 2015).

Targeting glucose uptake has already been followed for the treatment of tumors whose energy demands and resources are similar to those of inflammatory cells. The dependence on glycolysis of tumor cells was observed by Otto Warburg approximately 100 years ago and is known as the Warburg effect (Warburg, 1956). As glucose uptake is the key rate-limiting step of glycolysis, inhibiting glucose transporters (GLUTs) offers a promising therapeutic approach. GLUTs encompass a wide family of integral membrane proteins that are differentially expressed in various tissues. The development of GLUT inhibitors for the treatment of cancer has further gained momentum by the observation that ritonavir, which has originally been developed as an inhibitor of HIV protease, coincidentally inhibits the members 1 and 4 (Murata et al., 2000; Hertel et al., 2004; Vyas et al., 2010) of the GLUT family.

Preclinical studies *in vitro* and *in vivo* have shown that ritonavir alone and in combination with metformin inhibits proliferation of multiple myeloma and chronic lymphocytic leukemia cells, both of which exhibit abnormal glucose metabolism (Adekola et al., 2015; Dalva-Aydemir et al., 2015). The positive outcome of these experiments resulted in a first clinical trial to test metformin hydrochloride and ritonavir in patients with relapsing or refractory multiple myeloma or chronic lymphocytic leukemia (http://clinicaltrials.gov/ct2/show/NCT02948283).

Recently, we have developed a mouse model of UC, which is based on NOD-scid *IL-2Rγ*^null^ (NSG) mice reconstituted with peripheral blood mononuclear cells (PBMCs) from patients with UC (NSG-UC mice) (Palamides et al., 2016; Jodeleit et al., 2017; Al-amodi et al., 2017). In this model, the development of symptoms is induced by rectal challenge with ethanol and requires immune reconstitution with PBMCs from patients with active disease.

In light of the metabolic switch observed in UC patients, we evaluated ritonavir as a potential therapeutic for treatment of UC. First, the effect of ritonavir on CD4+ T cells was tested *in vitro*. Results indicate that ritonavir inhibited the activation of CD4+ T cells. Secondly, glutamic and aspartic acid were identified as potential biomarkers of inflammation in the NSG-UC mouse model. Finally, ritonavir was tested in the NSG-UC mouse model. Results indicate that ritonavir might be an attractive therapeutic for UC and that AAs are powerful biomarkers in this model.

## RESULTS

### Ritonavir affects subsets of CD4+ T cells

**Fig. 1.**
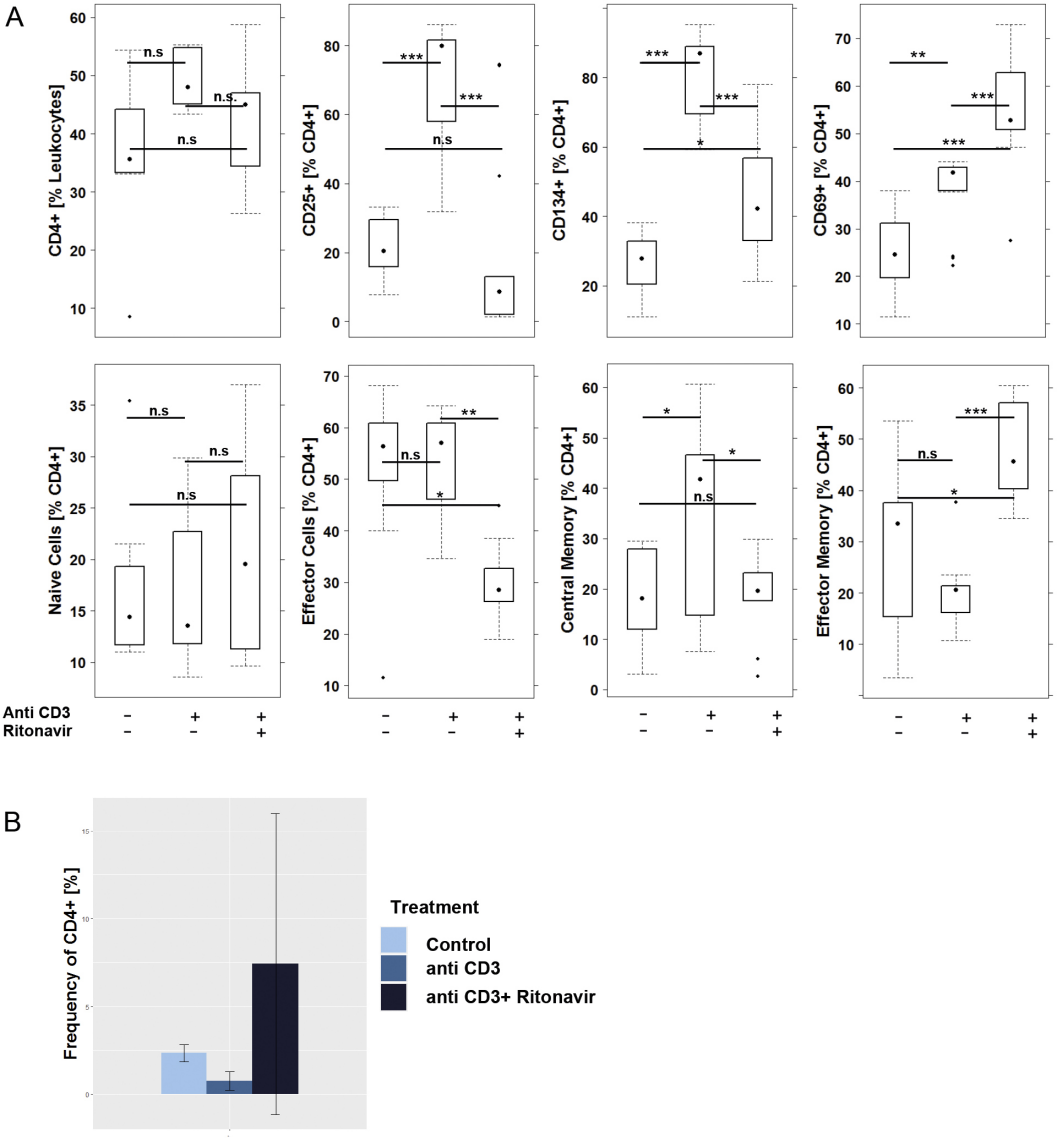
**Ritonavir affects frequencies of CD4+ T-cell subtypes.** Flow cytometric analysis of PBMCs that were incubated for 3 days in the presence or absence of anti-CD3 antibodies and ritonavir. Experiments were performed in triplicate. (A) Frequencies of CD4+ T cells and subtypes thereof depicted as boxplot diagrams (donor *n*=5). For comparison of groups, ANOVA followed by Tukey's HSD was conducted. Boxes represent upper and lower quartiles, whiskers represent variability and outliers are plotted as individual points (***P≤0.001, **P≤0.01, *P≤0.05, n.s., non-significant). Labels given on *x*-axes on the bottom row apply to all charts. (B) Frequencies of LIVE CD4+ cells depicted as barplots. Mean values are given; error bars are s.d. Results from two different donors are given.

Previous studies have shown that ritonavir inhibits proliferation and survival in multiple-myeloma and chronic-lymphocytic-leukemia cell lines *in vitro* and *in vivo* by shutting down the glycolytic pathway (Adekola et al., 2015; Dalva-Aydemir et al., 2015). However, these cell lines *per se* exhibit an abnormal glucose metabolism – a condition that we would not expect in unchallenged inflammatory cells. We therefore examined the response to ritonavir in subtypes of CD4+ T cells challenged with anti-CD3. PBMCs were isolated from five donors and experiments performed in triplicates. A total of 1×10^6^ cells were incubated with or without anti-CD3 antibodies for 72 h in the presence and absence of ritonavir and subjected to flow cytometric analysis as described in the Materials and Methods. (For a definition of cells and gating strategy, see Table S2 and Fig. S1). As shown in Fig. 1A and Table S3, frequencies of CD4+ cells increased in response to anti-CD3; however, this effect was not significant. Upon the addition of ritonavir, frequencies of CD4+ cells returned to levels of the control group, and the difference between the ritonavir-treated group and the anti-CD3-treated group was significant, whereas that between the ritonavir-treated group and the control group was not. Exposure to anti-CD3 antibodies resulted in significantly increased frequencies of CD25-, CD134- and CD69-expressing CD4+ cells, indicating a general activation of CD4+ T cells. Ritonavir reversed these effects with the exception of CD4+ CD69+ cells, whose frequencies even further increased. Challenge with anti-CD3 antibodies had no effect on naïve or effector CD4+ T cells, and only a minor effect on effector memory CD4+ T cells, whereas the frequencies of central memory CD4+ T cells increased significantly. Ritonavir had opposing effects on effector, central memory and effector memory CD4+ T cells. Ritonavir decreased frequencies of effector and central memory CD4+ T cells, whereas frequencies of effector memory cells increased. To ensure that ritonavir did not affect the viability of PBMCs, PBMCs from two different donors were analyzed for LIVE CD4+ and LIVE CD14+ cells using Zombie Green™ Fixable Viability Kit. As shown in Fig. 1B, the viability of CD4+ T cells was not impaired by ritonavir.

### Glutamic and aspartic acid are metabolic markers for inflammation in the NSG-UC mouse model

**Fig. 2.**
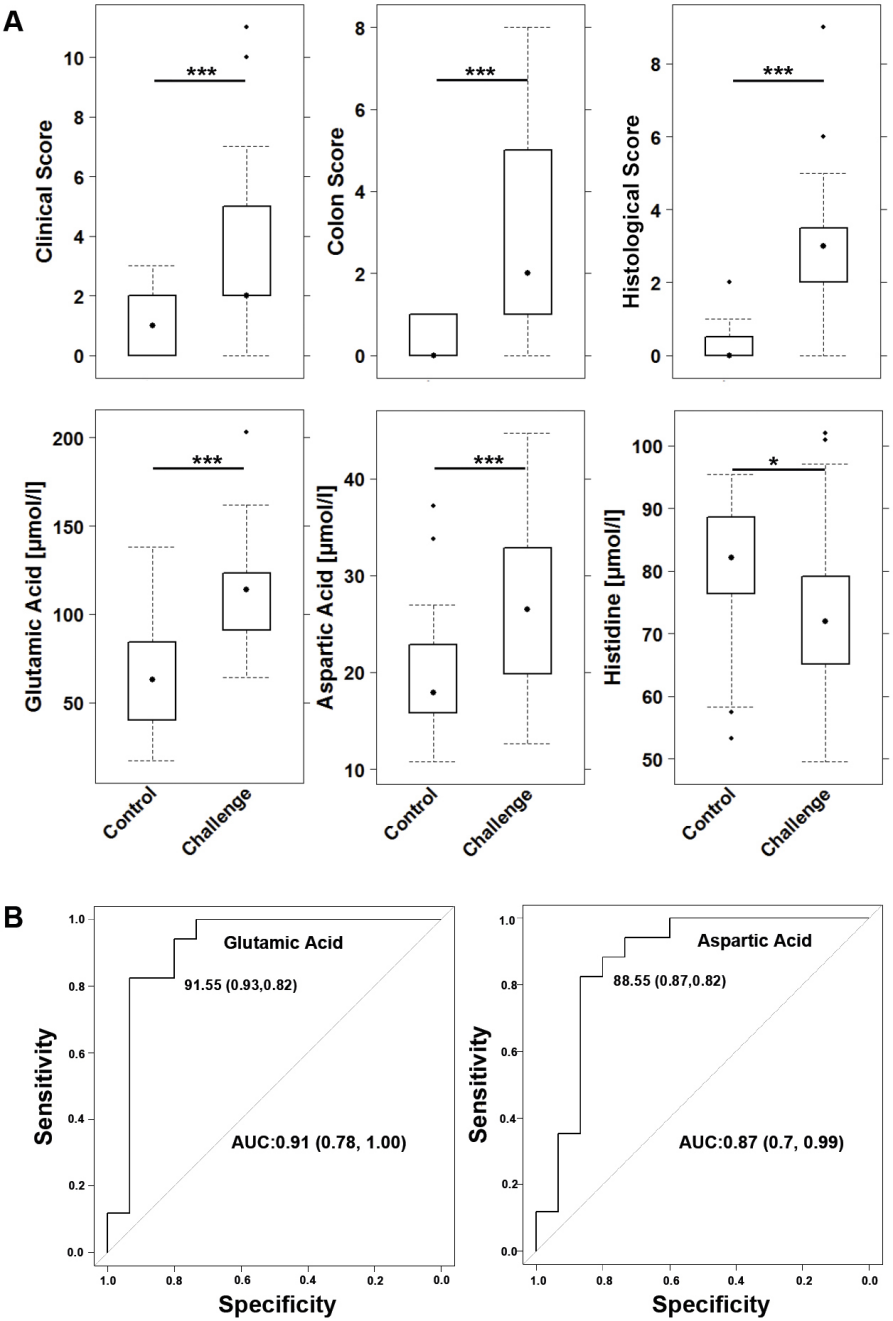
**Glutamic and aspartic acid are inflammatory markers in the NSG-UC mouse model.** NSG-UC mice were engrafted with PBMCs derived from UC patients, challenged with 10% ethanol at day 8, and 50% ethanol at days 15 and 18. (A) Amino acid (AA) levels and clinical and histological scores depicted as boxplot diagrams [donors *n*=3; unchallenged control group (control *n*=10); ethanol-challenged group (Challenge *n*=11)]. For comparison of groups, ANOVA followed by Tukey's HSD was conducted. Boxes represent upper and lower quartiles, whiskers represent variability and outliers are plotted as individual points (***P≤0.001, *P≤0.05). Labels given on *x*-axes on the bottom row apply to all charts. (B) ROC curve for glutamic and aspartic acid in discriminating control and challenged groups.

As previous studies have identified AAs as potential markers of inflammation in UC and Crohn's disease (Dawiskiba et al., 2014; Bjerrum et al., 2017), we measured AAs in serum of NSG-UC mice in response to challenge with ethanol. The study was performed as described previously (Palamides et al., 2016; Jodeleit et al., 2017). NSG mice were reconstituted with PBMCs and challenged according to a standard protocol as described in the Materials and Methods. At 8 days post-reconstitution, the mice were divided into two groups: one was left unchallenged (control), the other was challenged by rectal application of ethanol. Each group contained four to six animals and the experiment was repeated with four donors. Donors exhibited a simple clinical colitis activity index (SCCAI) (Walmsley et al., 1998) from one to seven. One donor was therapeutically naive, one was treated with mesalazine and vedolizumab, one with mesalazine, vedolizumab and glucocorticoids and one with glucticorticoids. Upon challenge with ethanol, mice stools became soft or liquid, the animals lost weight and the clinical activity score was elevated. Control animals displayed hardly any symptoms. Symptoms were classified according to a clinical score described in the Materials and Methods. Upon challenge, the clinical score increased significantly (Fig. 2A; for complete data set see Table S4). This observation was also corroborated by the colon score, which also increased significantly. As shown in previous studies (Palamides et al., 2016; Jodeleit et al., 2017; Al-amodi et al., 2017), histological analysis of the colon revealed the influx of a mixed infiltrate of leukocytes, edema, crypt loss and changes in the colon architecture (not shown). The histopathological manifestations were classified according to a histological score (Fig. 2A) and confirmed a significant response to ethanol. Prior to autopsy, serum samples were collected and subjected to AA analysis. Inflammation resulted in significantly elevated levels of glutamic and aspartic acid (Fig. 2A). Receiver operating characteristic (ROC) analysis identified both AAs as biomarkers with high potential to discriminate between challenged and unchallenged mice (glutamic acid: AUC=0.91; aspartic acid: AUC=0.87) (Fig. 2B). For further analysis, leukocytes were isolated from spleen and subjected to flow cytometric analysis as described in the Materials and Methods. Correlation analysis revealed a high correlation coefficient between both AAs and the clinical score, colon score and histological score, and with activated CD4+ CD69+ and CD4+ CD103+ T cells (Table 1), and thus corroborated the *in vitro* experiments. As observed in the human studies, levels of histidine declined upon challenge (Hisamatsu et al., 2015).

**Table 1.**
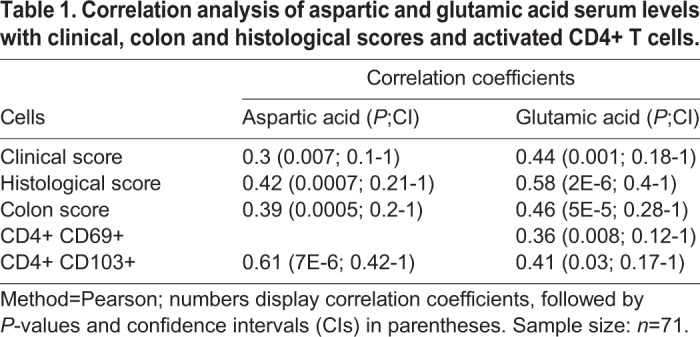
**Correlation analysis of aspartic and glutamic acid serum levels with clinical, colon and histological scores and activated CD4+ T cells.**

**Fig. 3.**
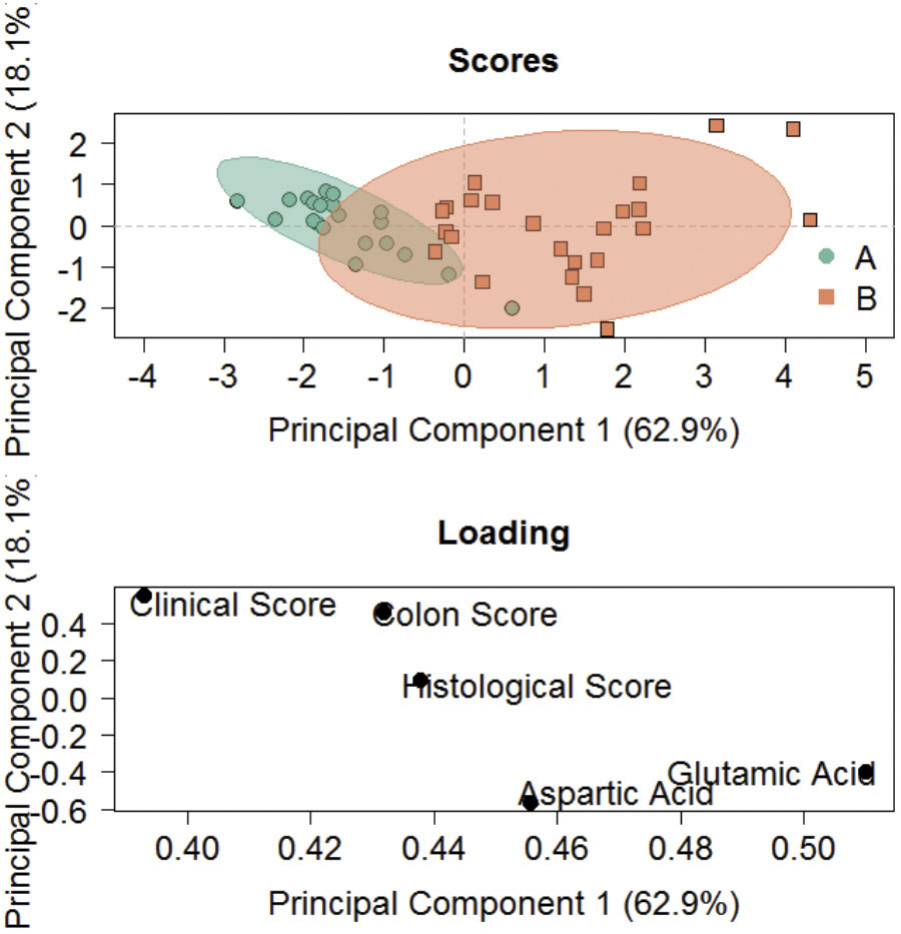
**PCA discriminates between control and ethanol-challenged group.** PCA score and loading plots (donors *n*=4, each group *n*=17). Sample size: donor *n*=4, (‘A’, green) control *n*=17, (‘B’, orange) challenge *n*=17. Ellipses display confidence areas of 0.95.

To further evaluate the combined power of the parameters to discriminate between the two groups, a principal component analysis (PCA) was performed to include the clinical, colon and histological scores, and levels of glutamic and aspartic acid. Scatter plots of PCA scores of single mice, with ellipses indicating confidence regions, depict mice of the control group as a closely clustered group, whereas ethanol-challenged mice are more distributed (Fig. 3). Multivariate regression analysis of the five parameters revealed an *R*^2^ predicted value of 0.62 and discriminating analysis based on partial least square that 95% of all predictions were correct.

### Treatment with ritonavir ameliorates disease phenotype in NSG-UC mouse model

**Fig. 4.**
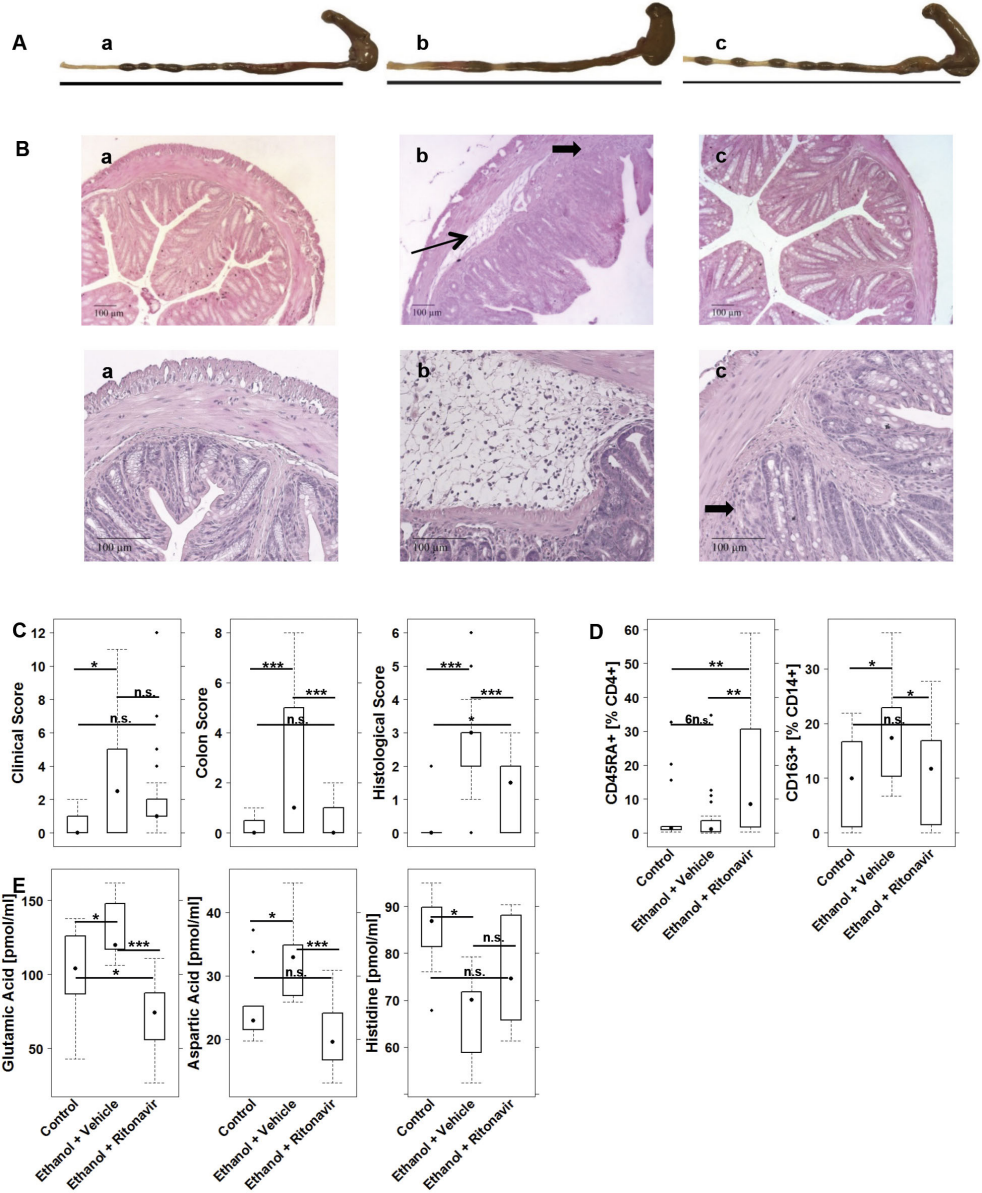
**NSG-UC mice benefit from treatment with ritonavir.** NSG-UC mice were engrafted with PBMCs derived from UC patients (*n*=4), challenged with 10% ethanol at day 8, and 50% ethanol at days 15 and 18, and treated with 20 mg/kg body weight ritonavir or carrier at days 7, 8 and 14-20. (a) Unchallenged control (Control *n*=15); (b) challenged control (Ethanol+Vehicle, *n*=21); (c) challenged and treated with ritonavir (Ethanol+Ritonavir, *n*=27). (A) Macrophotographs of colons at autopsy. (B) Photomicrographs of H&E-stained sections of distal parts of the colon from mice reconstituted with the same donor. Thin arrow indicates edema and influx of inflammatory cells; bold arrows indicate fibrosis. (C) Clinical, colon and histological scores of NSG-UC mice. (D) Frequencies of CD14+ subtypes isolated from spleens of mice. (E) Aspartic and glutamic acid serum levels depicted as boxplot diagrams (donor *n*=2, Control *n*=10, Ethanol+Vehicle *n*=10, Ethanol+Ritonavir *n*=11). For comparison of groups, ANOVA followed by Tukey's HSD was conducted. Boxes represent upper and lower quartiles, whiskers represent variability and outliers are plotted as individual points (***P≤0.001, **P≤0.01, *P≤0.05, n.s., non-significant). Labels given on *x*-axes on the bottom row apply to all charts.

To examine the effect of ritonavir *in vivo*, NSG-UC mice were treated with ritonavir. The study was performed as in the previous experiment; this time, however, the challenged group was treated with vehicle (ethanol+vehicle) and a third group was added consisting of mice challenged with ethanol and treated with ritonavir (ethanol+ritonavir). The study was repeated with PBMCs from four different UC patients (Table 2). (For the complete data set, please see Table S5.) The clinical score increased upon challenge and almost normalized with treatment of ritonavir (Fig. 4C). This observation was corroborated by macroscopic inspection of the colons. As shown in Fig. 4Ac, colons of ritonavir-treated mice appeared normal as compared to colons of ethanol-challenged mice (Fig. 4Ab), which exhibited the typical dilation and soft stools. Histological analysis further confirmed these results. Challenge with ethanol resulted in severe colon pathology to include crypt alterations, edema and fibrosis (Fig. 4Bb), whereas histological sections of ritonavir-treated mice appeared almost normal (Fig. 4Bc) (for scoring data, see Table S6). This observation was corroborated by the histological score, which was significantly reduced in the ritonavir-treated group (Fig. 4C).

**Table 2.**
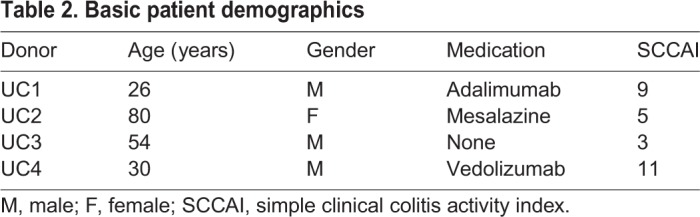
**Basic patient demographics**

As subtypes of monocytes had been previously identified as inflammatory markers in the NSG-UC mouse model (H.J., O.A.-A. and G.B. et al., unpublished), human leukocytes were isolated from the spleen of mice and subjected to flow cytometric analysis. The most profound effect of ritonavir was found on naïve CD4+ T cells and on M2 monocytes (CD14+ CD163+), whose frequencies were significantly elevated upon challenge with ethanol and reduced to normal levels in the presence of ritonavir (Fig. 4D). In contrast, frequencies of CD1a-expressing CD14+ monocytes were not significantly affected by treatment with ritonavir (Table S5). The anti-inflammatory effect of ritonavir was corroborated by analysis of AA levels. Glutamic and aspartic acids levels normalized upon treatment with ritonavir (Fig. 4E). Histidine levels increased upon treatment; however, this effect was not significant.

**Fig. 5.**
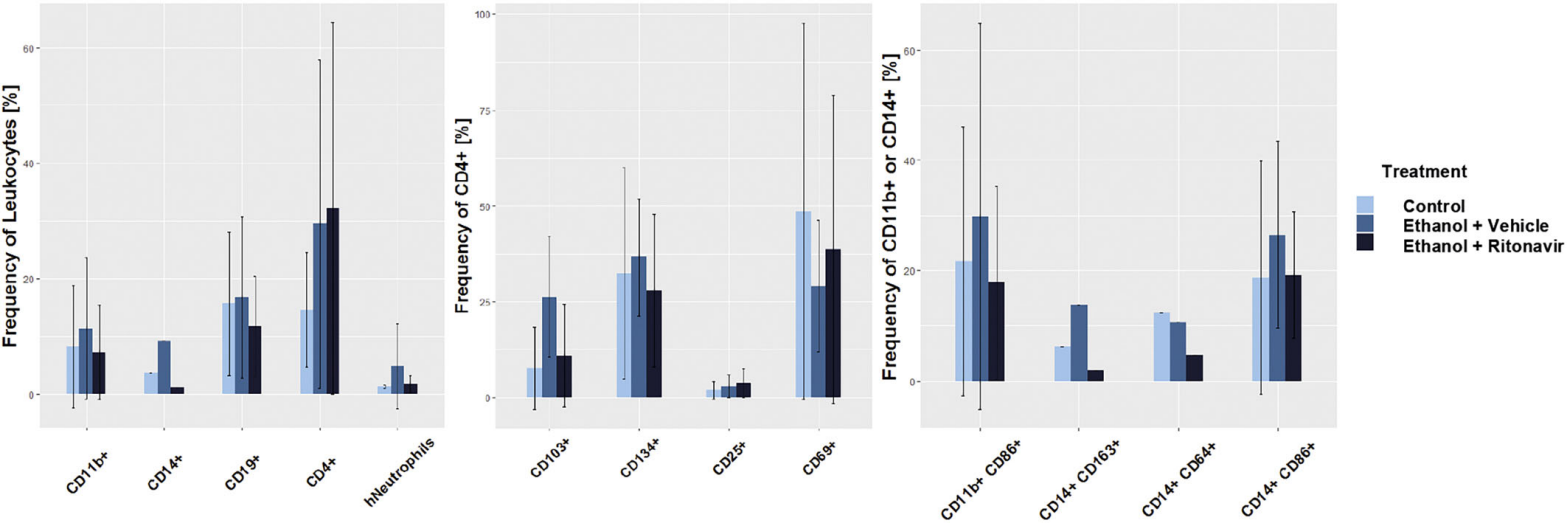
**Treatment with ritonavir affected frequencies of human leukocytes isolated from colon of NSG-UC mice.** Leukocytes were isolated from mice described in Fig. 4. Data were combined from four different experiments and samples from each group were pooled from six mice (Control *n*=4, Ethanol+Vehicle *n*=4, Ethanol+Ritonavir *n*=4). Mean values are given; error bars are s.d. Quantification was performed using flow cytometric analysis.

To examine the effect of ritonavir on inflammation in the compartment of the colon, leukocytes were isolated and subjected to flow cytometric analysis (Fig. 5). Frequencies of human CD11b+ macrophages, CD14+ monocytes, CD19+ B cells, and neutrophils were reduced upon treatment with ritonavir, whereas CD4+ T cells were unaffected (for gating strategy see Fig. S2). The strongest effect was observed on CD103- and CD134-expressing CD4+ T cells, mature CD11b+ macrophages and CD14+ monocytes, and M1 (CD14+ CD64+) and M2 (CD14+ CD163) monocytes; however, differences did not reach significance.

**Fig. 6.**
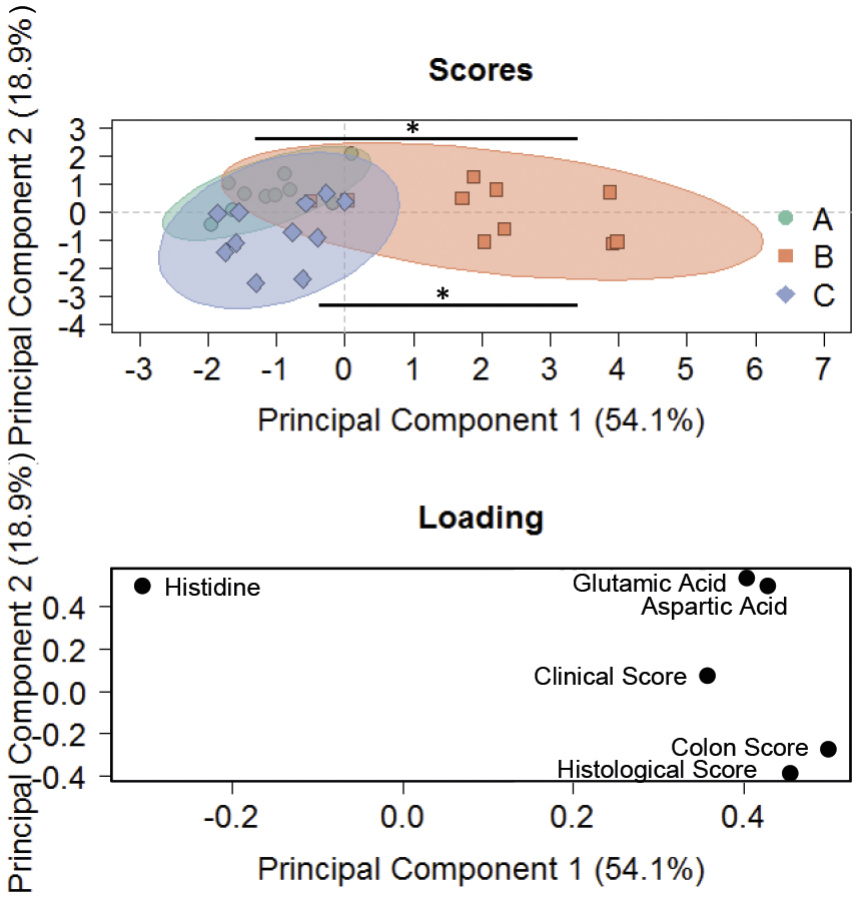
**PCA discriminates between the control group, ethanol-challenged group and ritonavir-treated group.** PCA scores and loading plots (‘A’, green=Control; ‘B’, orange=Ethanol+Vehicle; ‘C’, blue=Ethanol+Ritonavir). For comparison of groups, ordinal regression analysis was performed (*P≤0.05). Control group versus Ethanol+Vehicle *P*=0.02; Ethanol+PBS *P*=0.05.

As observed previously, PCA clearly segregated the challenged group from the control, and the treated group clustered with the control group when clinical score, histological score, and aspartic and glutamic acid levels were used as parameters (Fig. 6). Ordinal regression analysis revealed the significance of this model [Pr (Chi)=0.004], and these parameters resulted in a significant discrimination of the control group and the ethanol-challenged group (*P*=0.02), and the ethanol-challenged group and the ritonavir-treated group (*P*=0.05).

## DISCUSSION

The metabolic requirements of cancer cells differ from immune cells with regard to their capacity to modulate and adapt energy supply. Whereas cancer cells are continuously dependent on glycolysis due to their proliferative and migratory nature, immune cells acquire these traits upon challenge and modulate metabolic pathways accordingly. Therefore, the observation that activation of CD4+ T cells and the increase of frequencies of central memory CD4+ T cells in response to anti-CD3 were reversed by ritonavir did not come as a surprise. We did not expect, however, for ritonavir to increase the frequencies of effector memory cells. Central memory and effector memory cells differ with regard to expression of CD62L and CCR7, and, hence, ritonavir would impair the ability of memory cells to migrate to lymph nodes to be exposed to dendritic cells. We also did not expect that ritonavir increases the frequencies of early activated CD4+ CD69+ T cells. Further studies have to show which subtypes of cells are activated to understand the specific responses to ritonavir.

In the NSG-UC mouse model, the induction of inflammation had a significant effect on glutamic and aspartic acid, tryptophan and histidine levels, and corroborated results obtained in UC patients (Dawiskiba et al., 2014; Hisamatsu et al., 2015; Bjerrum et al., 2017). ROC analysis indicated that glutamic and aspartic acid were biological markers to discriminate with high sensitivity and specificity between unchallenged control and ethanol-challenged mice. PCA of the colon and histological scores, and glutamic and aspartic acid levels revealed a clustering of these two groups. This result is a major advantage as it most probably allows for evaluating more subtle differences in efficacy.

Mice benefitted significantly from treatment with ritonavir as indicated by the four parameters: clinical and histological scores and glutamic and aspartic acid levels in serum. PCA distinguished between control and challenged groups, and treatment with ritonavir resulted in clustering closer to the control group. Analysis of the human leukocytes isolated from spleen revealed that the most pronounced effect of ritonavir was observed in monocytes. Unlike in the *in vitro* experiments, ritonavir had no effect on activation of CD4+ T cells (data not shown).

An effect of ritonavir was, however, observed when human leukocytes isolated from colon were analyzed. As observed in the *in vitro* experiments, frequencies of activated CD4+ T cells declined with the exception of CD4+ CD69+ T cells. Likewise, frequencies of mature CD11b+ macrophages and CD14+ monocytes decreased, as well as M2 monocytes.

In summary, this study shows that inflammation in the NSG-UC mice has a significant metabolic effect in the NSG-UC model, which partially reflects the metabolic effects observed in UC patients. AA levels are potential biological markers of inflammation so might improve the evaluation of efficacy of therapeutics. The positive outcome of the preclinical study in the NSG-UC model suggests that UC patients might benefit from adjuvant treatment with ritonavir during times of active disease, and might encourage the development of novel GLUT inhibitors.

## MATERIALS AND METHODS

### Ethical considerations

All donors gave informed written consent and the study was approved by the Institutional Review Board (IRB) of the Medical Faculty at the University of Munich (2015-22).

Animal studies were approved by the ethics committee of the government of Upper Bavaria, Germany (55.2-1-54-2532-76-15) and performed in compliance with German Animal Welfare Laws.

### Isolation of PBMCs and engraftment

Isolation of PBMCs and engraftment was performed as described previously (Jodeleit et al., 2017). Sixty milliliters of peripheral blood were collected in trisodium citrate solution (S-Monovette, Sarstedt, Nürnberg, Germany) from the arm vein of patients suffering from UC or healthy control subjects. The blood was diluted with Hank's balanced salt solution (HBSS, Sigma-Aldrich, Deisenhofen, Germany) in a 1:2 ratio and 30 ml of the suspension were loaded onto LeucoSep tubes (Greiner Bio One, Frickenhausen, Germany). PBMCs were separated by centrifugation at 400 ***g*** for 30 min and no acceleration. The interphase was extracted and diluted with phosphate buffered saline (PBS) to a final volume of 40 ml. Cells were counted and centrifuged at 1400 ***g*** for 5 min. The cell pellet was resuspended in PBS at a concentration of 4×10^6^ cells in 100 µl.

Six- to eight-week-old NSG mice were engrafted with 100 µl cell suspension into the tail vein on day 1.

### Cell culture

PBMCs of healthy individuals and UC patients were isolated. The cell pellet was resuspended in RPMI (Thermo Fisher Scientific, Waltham, MA, USA) at a concentration of 1×10^6^ cells/ml. Additionally, 500 µl RPMI with 10% FCS and 1% penicillin-streptomycin (Sigma-Aldrich, St Louis, MO, USA) were added to each well and sample. Wells containing PBMCs and RPMI or PBMCs, RPMI and anti-CD3 (1 µg/ml, BioLegend, San Diego, CA, USA) served as negative and positive controls, respectively. Ritonavir was dissolved in 100% ethanol (10 mg/ml). For analysis of the effect of ritonavir, 100 µg/ml ritonavir (Sigma-Aldrich, Deisenhofen, Germany) was added. Cells were incubated for 72 h. The content of each well was centrifuged at 1400 ***g*** for 5 min. The pellet was resuspended in FACS buffer and labeled for flow cytometry.

To differentiate between live and dead cells, staining with Zombie Green™ Fixable Viability Kit (BioLegend) was performed according to the manufacturer's instructions.

### Animal study protocol

NOD-scid *IL-2Rγ*^null^ (NSG) mice were obtained from Charles River Laboratories (Sulzfeld, Germany). Mice were kept under specific pathogen-free conditions in individually ventilated cages in a facility controlled according to the Federation of Laboratory Animal Science Association (FELASA) guidelines. Following engraftment (day 1), mice were pre-sensitized by rectal application of 150 µl of 10% ethanol on day 8 using a 1 mm cat catheter (Henry Schein, Hamburg, Germany), which is normally used for urinary catheterization in cats. In our case, we used it for rectal application of ethanol because of a similar diameter of a mouse rectum and a cat's urethra. The catheter was lubricated with Xylocain^©^Gel 2% (AstraZeneca, Wedel, Germany). Rectal application was performed under general anesthesia using 4% isofluran. Following application, mice were kept at an angle of 30° to avoid ethanol dripping. On days 15 and 18, mice were challenged by rectal application of 50% ethanol following the protocol of day 8. Mice were sacrificed by cervical dislocation on day 21.

Ritonavir was dissolved in 100% ethanol (100 mg/ml) and diluted with PBS to a final concentration of 5 mg/ml of 5% ethanol. Mice were treated by intraperitoneal application of 500 µg or 5% ethanol in PBS (vehicle) on days 7, 8 and 14-20. The dosage corresponds to 20 mg/kg body weight, which is recommended for maintenance therapy of HIV patients and calculated for a 25 g mouse.

### Clinical activity score

The assessment of severity of colitis was performed daily according to the following scoring system: loss of body weight: 0% (0), 0-5% (1), 5-10% (2), 10-15% (3), 15-20% (4). Stool consistency: formed pellet (0), loose stool or unformed pellet (2), liquid stools (4). Behavior: normal (0), reduced activity (1), apathy (4) and ruffled fur (1). Body posture: intermediately hunched posture (1), permanently hunched posture (4). The scores were added daily into a total score with a maximum of 18 points per day. Animals who suffered from weight loss >20%, rectal bleeding, rectal prolapse, self-isolation or a severity score >7 were euthanized immediately and not taken into count.

### Histological analysis

Samples from distal parts of the colon were fixed in 4% formaldehyde for 24 h before storage in 70% ethanol and were routinely embedded in paraffin. Samples were cut into 3-µm sections and stained with hematoxylin and eosin (H&E). Epithelial erosions were scored as follows: no lesions (0), focal lesions (1), multifocal lesions (2), major damage with involvement of basal membrane (3). Inflammation was scored as follows: infiltration of few inflammatory cells into the lamina propria (1), major infiltration of inflammatory cells into the lamina propria (2), confluent infiltration of inflammatory cells into the lamina propria (3), infiltration of inflammatory cells including tunica muscularis (4). Fibrosis was scored as follows: focal fibrosis (1), multifocal fibrosis and crypt atrophy (2). The presence of edema – minimal (1), moderate (2), severe (3), hyperemia (1) and crypt abscess (1) – was scored with additional points in each case. The scores for each criterion were added to give a total score ranging from 0 to 12. Images were taken with a Zeiss AxioVert 40 CFL camera. Figures show representative longitudinal sections in original magnification. In Adobe Photoshop CS6, a tonal correction was applied in order to enhance contrasts within the pictures.

### Isolation of human leukocytes

To isolate human leukocytes from murine spleen, spleens were minced and cells filtrated through a 70 µl cell strainer (Greiner Bio-One, Frickenhausen, Germany) followed by centrifugation at 1400 ***g*** for 5 min and resuspended in FACS buffer (1× PBS, 2 mM EDTA, 2% FCS) (Jodeleit et al., 2017). For further purification, cell suspensions were filtrated using a 35-µm cell strainer (Greiner Bio-One) and then labeled for flow cytometry analysis.

### Isolation of LPMCs

For isolation of lamina propria mononuclear cells (LPMCs), a modified protocol of Weigmann et al. (2007) was used. The washed and minced colon was pre-digested for 20 min in pre-digestion solution containing 1× HBSS (Thermo Scientific, Darmstadt, Germany), 5 mM EDTA, 5% FCS, 100 U/ml penicillin-streptomycin (Sigma-Aldrich) in an orbital shaker with slow rotation (40 ***g***) at 37°C. Epithelial cells were removed by filtering through a nylon filter. Following washing with PBS, the remaining colon pieces were digested twice for 20 min in digestion solution containing 1× RPMI (Thermo Scientific), 10% FCS, 1 mg/ml collagenase A (Sigma-Aldrich), 10 KU/ml DNase I (Sigma-Aldrich) and 100 U/ml penicillin-streptomycin (Sigma-Aldrich) in an orbital shaker with slow rotation (40 ***g***) at 37°C (Weigmann et al., 2007). Isolated LPMCs were collected by centrifugation at 1400 ***g*** for 5 min and resuspended for FACS analysis. Cell suspensions were filtrated one more time using a 35-μm cell strainer for further purification before labeling the cells for flow cytometry analysis.

### Flow cytometry analysis

Labeling of human leukocytes was performed according to Table S1. All antibodies were purchased from BioLegend and used according to the manufacturer's instructions. Antibodies were diluted 1:200. Flow cytometry was performed using a BD FACS Canto II™ and analyzed with FlowJo 10.1 software (FlowJo LLC, OR, USA). Cells were quantified according to the gating strategy depicted in Fig. S1.

### Detection of amino acids

Samples were prepared according to the manufacturer's instructions. Following incubation of 100 µl of serum with internal standards for 5 min, 25 µl of 15% 5-sulfosalicylic acid was added and samples were centrifuged at 9000 ***g*** for 15 min at 4°C. Supernatants were filtered through a 0.2-µm membrane and 75 µl of lithium loading buffer was added. Samples were analyzed by the AA analyzer Biochrom 30+ (Biochrom Ltd, Cambridge, UK).

### Statistics

All statistical analysis was performed with R: A language and environment for statistical computing (R Foundation for Statistical Computing, Vienna, Austria; https://www.R-project.org/) and BRB Array Tools (https://brb.nci.nih.gov/BRB-ArrayTools/). Variables were represented with mean, standard deviation and median values. A two-sided Student's *t*-test and a confidence level=0.95 was used to compare binary groups and, for more than two groups, ANOVA followed by Tukey’s HSD was conducted. For correlation analysis, Pearson correlation was used. To minimize the risk of overfitting, multivariate regression models were performed applying the kernelpls fit method and performing a leave one out cross validation procedure. For PCA, a confidence interval of 0.95% was used.

## Supplementary Material

Supplementary information
