# Supplementary material for: Targeting ulcerative colitis by suppressing glucose uptake with ritonavir
Source: Dis Model Mech. 2018 Nov 21;11(11):dmm036210. doi: 10.1242/dmm.036210 (PMC6262818; doi:10.1242/dmm.036210)
Supplement: Supplementary information [file dmm-11-036210-s1.pdf]

A

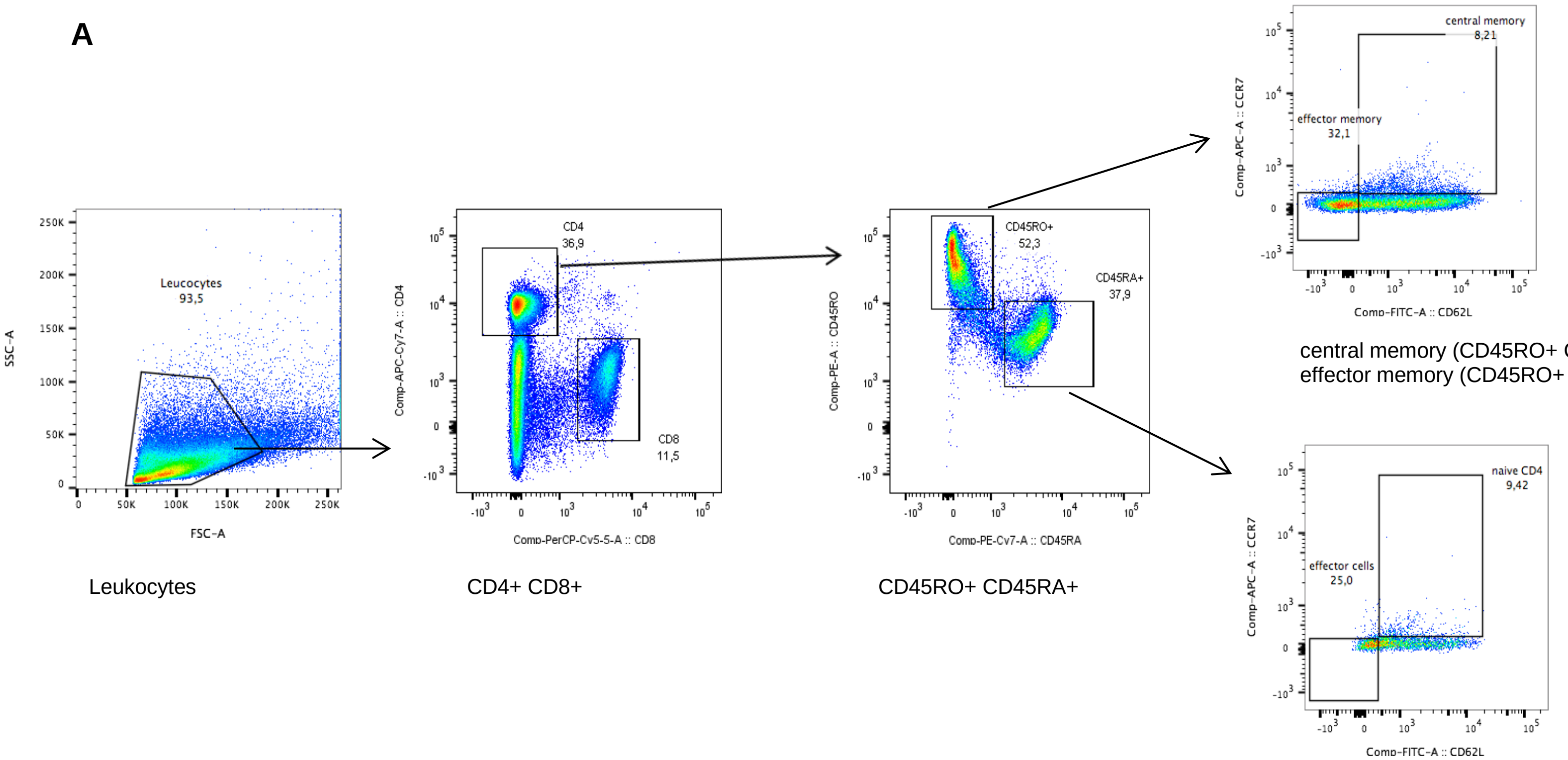

Leukocytes

CD4+ CD8+

CD45RO+ CD45RA+

central memory (CD45RO+ CCR7+ 62L+)
effector memory (CD45RO+ CCR7- 62L-)

naive CD4 (CD45RA+ CCR7+ 62L+)
effector cells (CD45RA+ CCR7- 62L-)

Disease Models & Mechanisms • Supplementary information

B

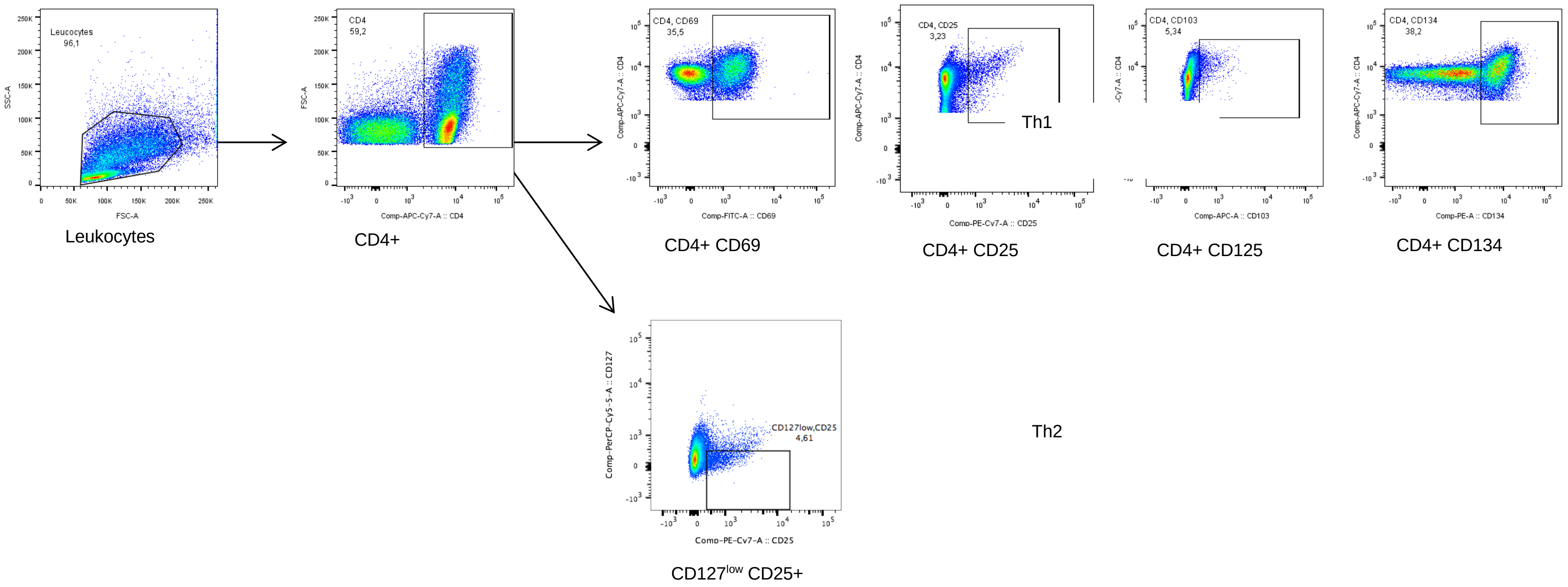

Disease Models & Mechanisms • Supplementary information

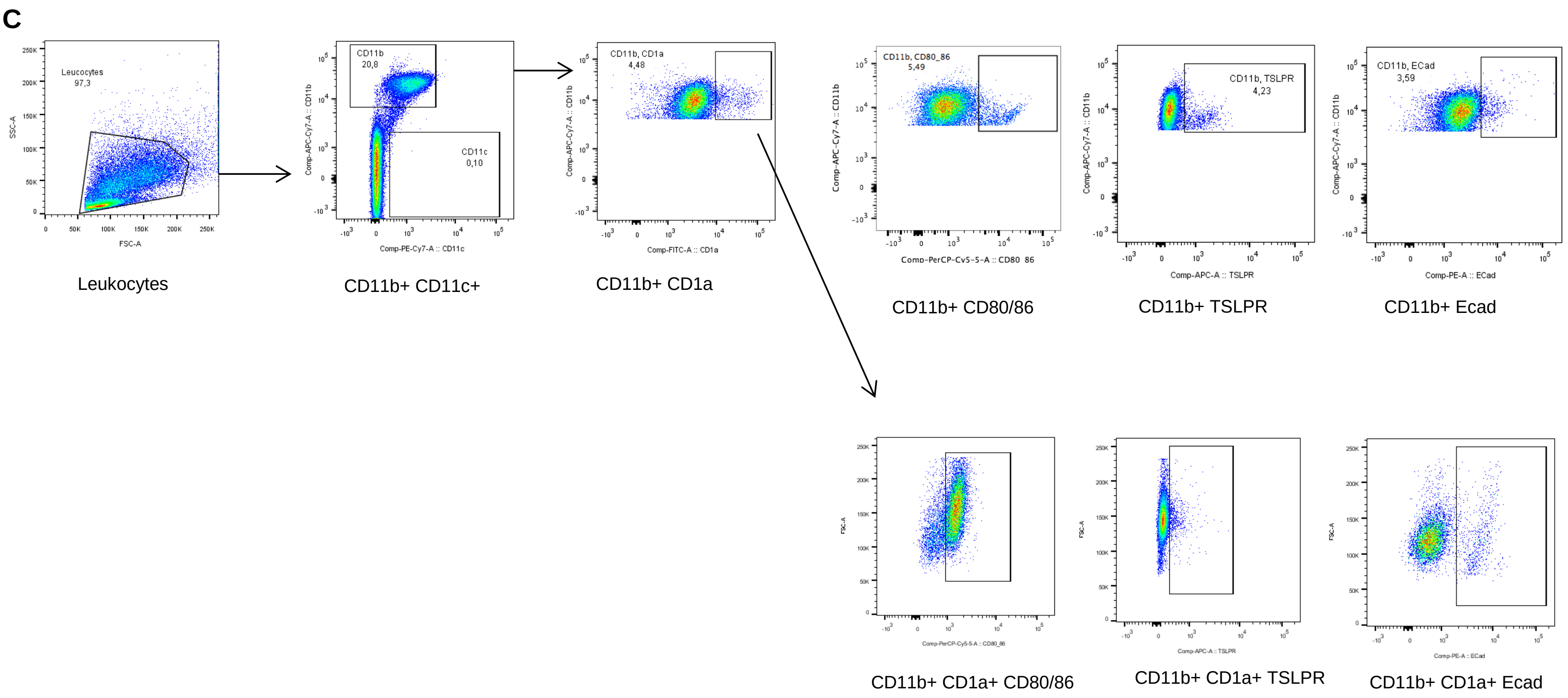

Disease Models & Mechanisms • Supplementary information

D

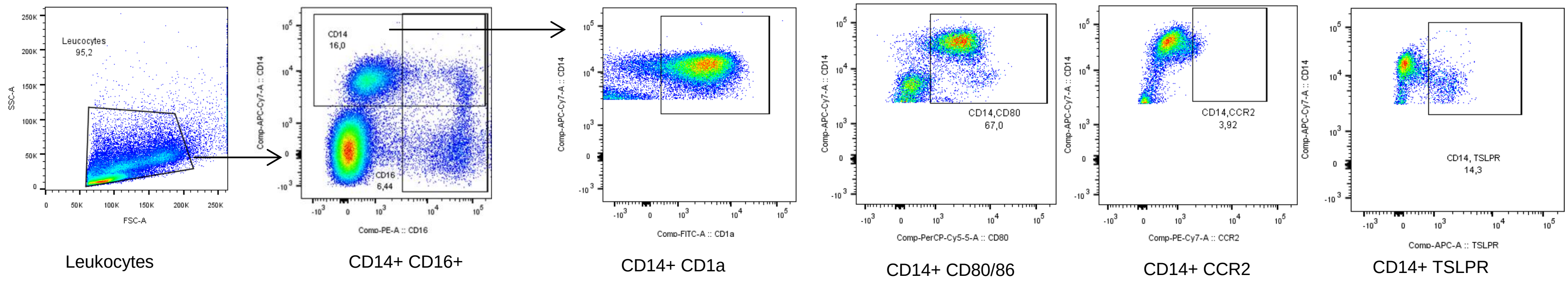

Figure S1. Gating strategy (A) central memory, effector memory, naive and effector CD4+ cells; (B) Th1, Th2, Th17 and Th22 T-cells; (C) activated CD4+ T-cells and Tregs; (D) CD11b+ macrophages; (E) CD14+ monocytes. Leukocytes were labeled as described in Material and Methods using antibodies listed in Table S1**.**

Disease Models & Mechanisms • Supplementary information

A

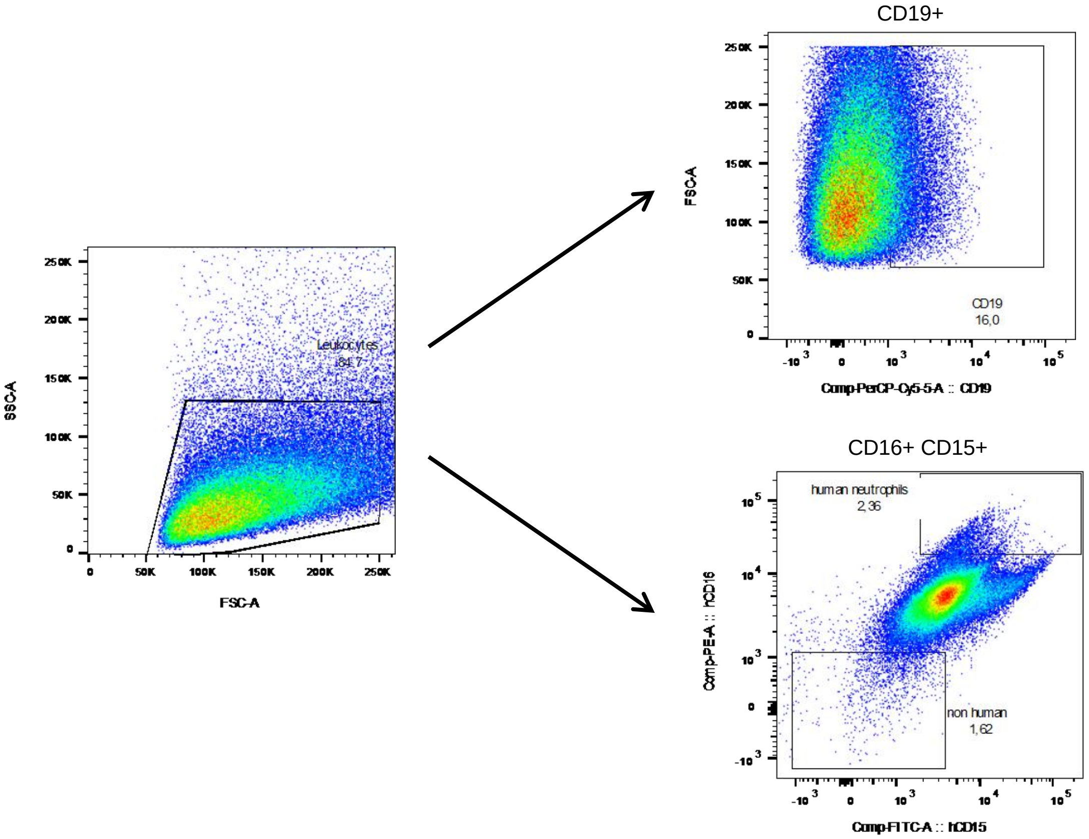

Disease Models & Mechanisms • Supplementary information

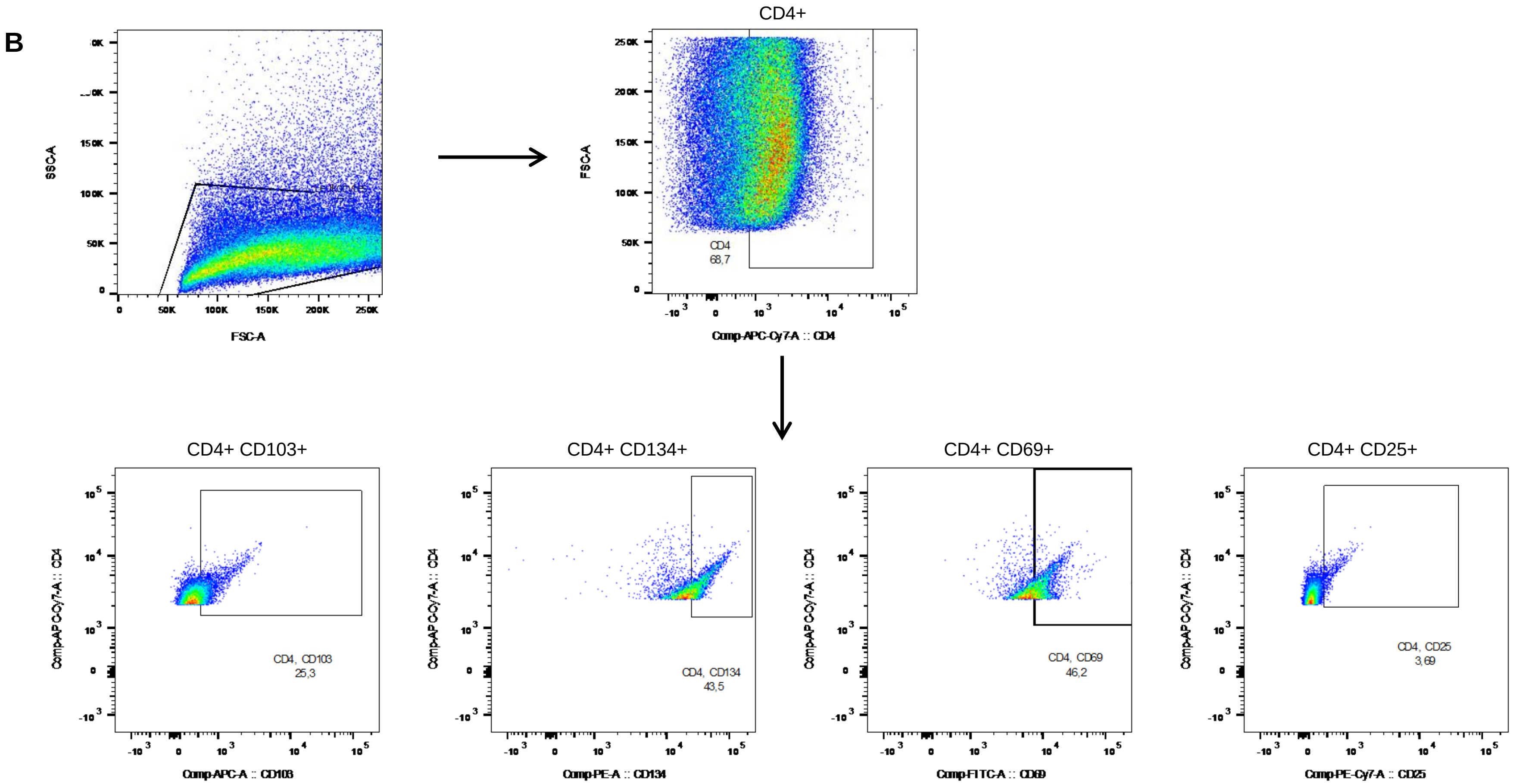

Disease Models & Mechanisms • Supplementary information

C

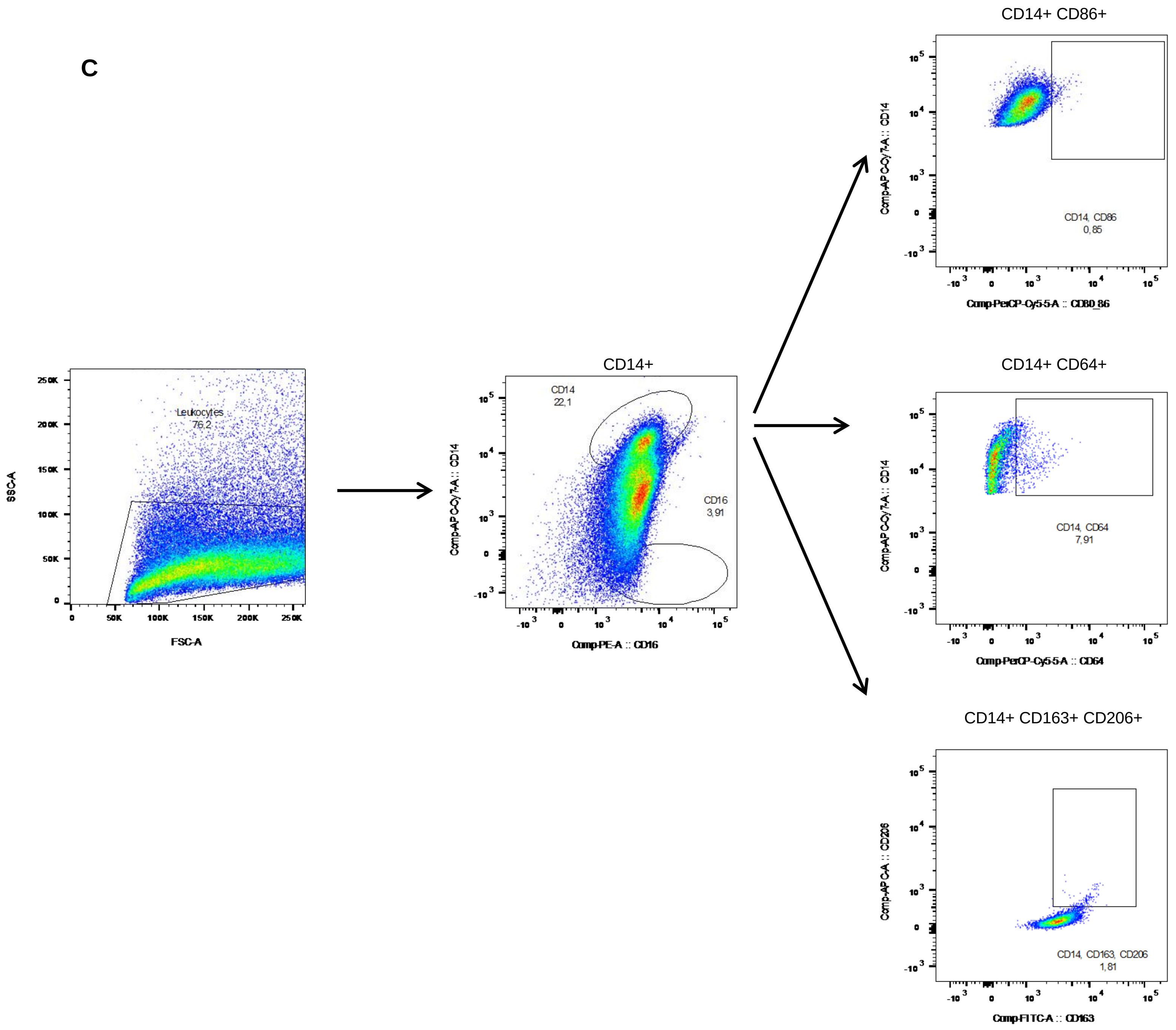

Disease Models & Mechanisms • Supplementary information

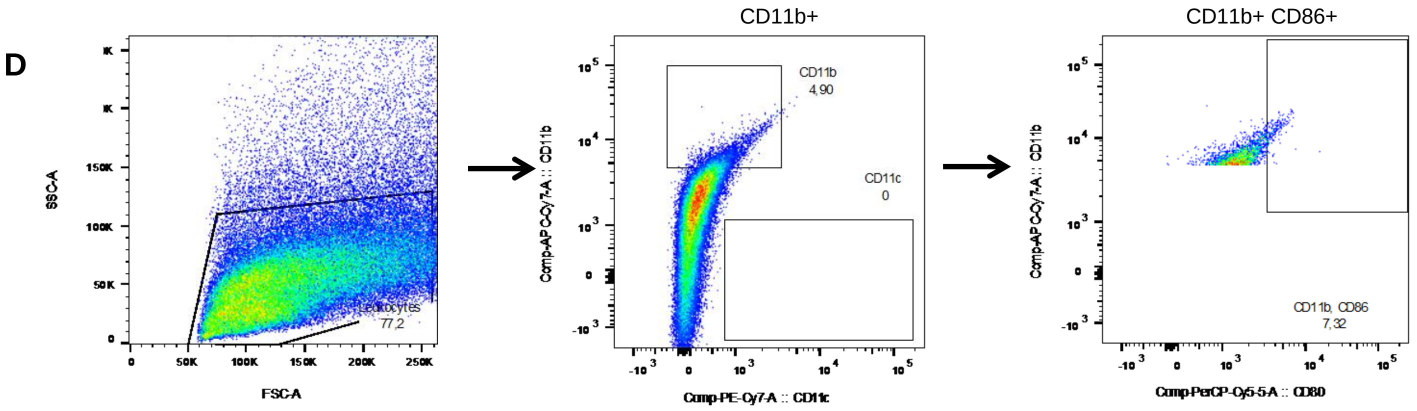

Figure S2. Gating strategy for leukocytes isolated from colon. (A) B cells, human neutrophiles; (B) activated CD4+ T-cells; (C) CD14+ monocytes (D) CD11b+ macrophages. Leukocytes were labeled as described in Material and Methods using antibodies listed in Table S1**.**

Disease Models & Mechanisms • Supplementary information

Table S1. Antibodies used to label leucocytes

Surfacemarker (anti human)	Colour	Clone	Catalogue Number
CD19	Peridine-chlorophyll-protein complex cyanine dye (PerCP-Cy™ 5.5)	HIB19	302230
CD38	Phycoerythrin (PE)	HB-7	356603
CD27	Pe-Cy7	M-T271	356411
IgD	Fluorescin isothiocyanate (FITC)	IA6-2	348217
CD4	Allophycocyanin (APC)- Cy7	OKT4	317417
CD62L	FITC	DREG-56	304803
CD45RO	PE	UCHL1	304205
CD45RA	Pe-Cy7	HI100	304125
CCR7	APC	G043H7	353213
CD8	PerCP-Cy™ 5.5	HIT8a	300923
CD103	APC	Ber-ACT8	350215
CD25	PE-Cy7	BC96	302611
CD14	APC-Cy7	HCD14	325619
CCR2	PE-Cy7	K036C2	357211
CD80/86	PerCP-Cy™ 5.5	IT2.2	305419
TSLPR*	APC	1B4	322807
CD1a (biotin)/secondary Ab streptavidin	FITC	HI149	300112
CD64	PerCP-Cy™ 5.5	10.1	305023
CD163	FITC	GHI/61	333617
CD206	APC	15-2	321109
CD16	PE	3G8	302007
CD11b	APC-Cy7	ICRF44	301341
CD11c	PE-Cy7	3.9	301607
ECad	PerCP-Cy™ 5.5	67A4	324105
CD127	PerCP-Cy™ 5.5	A019D5	351321
CD69	FITC	FN50	310903
CD134 (Ox40)	PE	Ber-ACT35	350003
CXCR3	FITC	G025H7	353703
CCR4	PE	L291H4	359411
CCR6	PE-Cy7	G034E3	353417
CCR10	APC	6588-5	341505

* Thymic stromal lymphopoietin receptor

Table S2. Cellular markers used to define immune cells

Marker	Definition
CD19+ CD27+ IgD+	Unswitched memory B-cell
CD19+ CD27+ IgD-	Switched memory B-cell
CD19+ CD38+	Plasma cell
CD4+ CD45RO+ CD62L- CCR7-	Effector memory CD4+ T- cell
CD4+ CD45RO+ CD62L+, CCR7+	Central memory
CD4+ CD103+	Mucosal regulatory CD4+ T cell
CD4+ CCR4+	Th2, effector regulatory T- cell
CD4+ CD25+ CD127-	Regulatory T-cell
CD4+ CD134+	Activated CD4+ T cell
CD4+ CD69+	Activated CD4+ T cell
CD4+ CD25+	Activated CD4+ T cell
CD14+	Monocytes (MC)
CD14+ TSLPR*+	MC, expressing TSLPR
CD14+ CD64+	M1MC
CD14+ CD163+ CD206+	M2 MC, scavenging cells
CD14+ CD1a	MC CD1a expressing
CD11b+	Conventional dendritic cells (cDC1)
CD11b+ CD80/86+	cDC1, mature
CD11b+ HLADR+	cDC1, presenting
CD11b+ TSLPR*+	cDC1 TSLPR expressing
CD11b+ CD1a+	cDC1 CD1a expressing

* Thymic stromal lymphopoietin receptor

Disease Models & Mechanisms • Supplementary information

Table S3. Complete data set from *in vitro* studies

Click here to Download Table S3

Table S4. Complete data set from animal study

Click here to Download Table S4

Table S5. Complete data set from animal study testing ritonvir

Click here to Download Table S5

Table S6.

Click here to Download Table S6
